# Amplitude of Low Frequency Fluctuation (ALFF) in the Cervical Spinal Cord with Stenosis: A Resting State fMRI Study

**DOI:** 10.1371/journal.pone.0167279

**Published:** 2016-12-01

**Authors:** Xiaojia Liu, Wenshu Qian, Richu Jin, Xiang Li, Keith DK Luk, Ed. X. Wu, Yong Hu

**Affiliations:** 1 Department of Orthopaedics and Traumatology, Li Ka Shing Faculty of Medicine, The University of Hong Kong, Pokfulam, Hong Kong; 2 Department of Electrical and Electronic Engineering, Faculty of Engineering, The University of Hong Kong, Pokfulam, Hong Kong; 3 Spinal division, Department of Orthopaedics, Affiliated Hospital of Guangdong Medical University, Guangdong, China; Universita degli Studi di Palermo, ITALY

## Abstract

Cervical spondylotic myelopathy (CSM) is a common spinal cord dysfunction disease with complex symptoms in clinical presentation. Resting state fMRI (rsfMRI) has been introduced to study the mechanism of neural development of CSM. However, most of those studies focused on intrinsic functional connectivity rather than intrinsic regional neural activity level which is also frequently analyzed in rsfMRI studies. Thus, this study aims to explore whether the level of neural activity changes on the myelopathic cervical cord and evaluate the possible relationship between this change and clinical symptoms through amplitude of low frequency fluctuation (ALFF). Eighteen CSM patients and twenty five healthy subjects participated in rsfMRI scanning. ALFF was investigated on each patient and subject. The results suggested that ALFF values were higher in the CSM patients at all cervical segments, compared to the healthy controls. The severity of myelopathy was associated with the increase of ALFF. This finding would enrich our understanding on the neural development mechanism of CSM.

## Introduction

Cervical spondylotic myelopathy (CSM) is the most common disease resulting in spinal dysfunction [1]. The degeneration of cervical spine causes stenosis of the spinal canal leading to static and dynamic compression to the cervical cord [2]. However, this simple mechanistic explanation cannot explain the variety of clinical presentation because the neurological function deficits are not always associated with the severity of stenosis [3]. As a result, the mechanism of neural development of CSM is still obscure due to lack of more detailed information.

To obtain more information, many neuroimaging techniques have been introduced to explore the mechanism of neural development of CSM in different aspects. Anatomic magnetic resonance imaging (MRI) can visualize the cervical spondylosis, malalignment, cord compression and high signal intensity within the cord [4, 5]. However, observation of cord compression degree is usually inconsistent with the severity of CSM symptoms in clinical practice [6–9].

Resting state fMRI (rsfMRI) has advantages over anatomic MRI in exploring pathology of neural diseases. RsfMRI focused on low-frequency blood oxygen level-dependent (BOLD) fluctuations which are considered to reflect intrinsic neural activity. In recent years, rsfMRI has already been introduced in spinal cord studies and revealed that the spinal cord is intrinsically organized [10–12].

To observe intrinsic functional neural activity, one main stream of rsfMRI is functional connectivity which focuses on temporal correlation of BOLD signal to reflect neuronal synchronization [13–15]. A primate spinal cord injury model proposed in one study has once suggested that the intrinsic functional connectivity within the spinal cord was altered after injury [16]. However, functional connectivity is useful in revealing relationship among different regions of the neural system but unable to explore specific regional neural activity dynamics.

Another rsfMRI analysis that focuses on the amplitude of BOLD signal can be applied to analyze specific regional neural activity dynamics. Amplitude of low frequency fluctuation (ALFF) is one of the most reliable and reproducible rsfMRI parameters reflecting the level of regional functional neural activity [17–22] and may help us to explain the neural development mechanism of CSM.

In this study, rsfMRI was employed to investigate the regional functional neural activity strength in both healthy subjects and CSM patients. ALFF values were calculated and compared between healthy subjects and CSM patients.

The aim of this study is to explore whether the ALFF will change after CSM. Based on previous studies, we hypothesized that (1) CSM patients would present ALFF alteration on the spinal cord, and (2) these alterations of ALFF would correlate with clinical CSM evaluating scores.

## Material and Methods

### Subjects Information

Ethical approval for the study was granted by the Clinical Research Ethics approved by the Institutional Review Board of the University of Hong Kong/Hospital Authority (UW 12–468). The written consent was obtained from every participant before he/she join this study. The procedure for introduction and consent was approved by the ethics committees. A total of 25 healthy subjects (female/male = 7/18, age = 32±9 years, age ranged from 22 to 61 years) and 18 CSM patients (female/male = 5/13, age = 58±14 years, age ranged from 26 to 83 years, duration of symptom > 1 year, compression position range from C3 to C6 segment) were recruited in this study, and informed consents were obtained from all participants.

For healthy subjects, the inclusion criteria were that subjects should be normal sensory and motor functions and negative Hoffman’s sign. Those subjects with past history of spinal cord injury, myelopathy, other neurological diseases or narrowing of the spine canal on T2 weighted (T2W) images were excluded.

All the patients with CSM were examined and diagnosed by experienced spine surgeons with typical manifestation and imaging findings. The inclusion criteria of CSM in this study were: 1) the presentation of spinal canal stenosis in T2W images measured by anterior-posterior compression ratio (APCR); 2) increased signal intensities within the spinal cord observed on the sagittal plane of T2W images. Patients with acute spinal cord injuries, prior spinal intervention, other co-existing neurological disorders (e.g., multiple sclerosis) or claustrophobia were excluded. The neurological deficits of all the CSM patients were evaluated by Japanese Orthopaedic Association (JOA) score system.

### Anatomic MRI

To investigate the degree of cord compression of the CSM patients, T2W MRI images on the axial plane were acquired for each patient through a fast spin echo sequence and APCR was calculated for each body and disc level from vertebrae C2 to C7. APCR was calculated in the following process: transverse diameter (a) and anterior-posterior diameter (b) of spinal cord were measured on the cross section of T2W images; APCR = b/a [3]. The smallest value of APCR was selected to represent the severity of cord compression.

### Resting state fMRI data acquisition and preprocessing

RsfMRI data acquisition was conducted by a 3 Tesla (Philips Achieva, Amsterdam, the Netherlands) whole body MRI scanner and a 4-channel neurovascular coil. The rsfMRI images were acquired through a gradient-echo echo planar imaging (GE-EPI) sequence with the following parameters: repetition time (TR)/echo time (TE) = 2000/30 ms, number of slices = 26, voxel size = 1.25 × 1.25 × 4 mm, field of view (FOV) = 80 × 80 × 104 mm (from the level of vertebrae C1 to C7). The sequence is typically used for brain while the parameters were optimized for the spinal cord. Through the Statistical Parametric Mapping 8 (SPM8, http://www.fil.ion.ucl.ac.uk/spm/) and Resting-State fMRI Data Analysis Toolkit (REST, http://restfmri.net/forum/index.php) toolboxes in MATLAB 7.11.0 (Mathworks, Natick, MA, USA), the raw EPI images were processed for slice timing, motion correction, nuisance regression, detrend, and band-pass filtering (0.01–0.08 Hz). Regressors included: 1) cerebrospinal fluid (CSF) pulsation signal and tissue motion signal that was extracted through independent component analysis (ICA) through gift (http://mialab.mrn.org/software/gift/) toolbox; 2) motion correction parameters (x and y translation)[12]. The first 10 volumes were discarded to exclude the initial transient effects.

**Fig 1:**
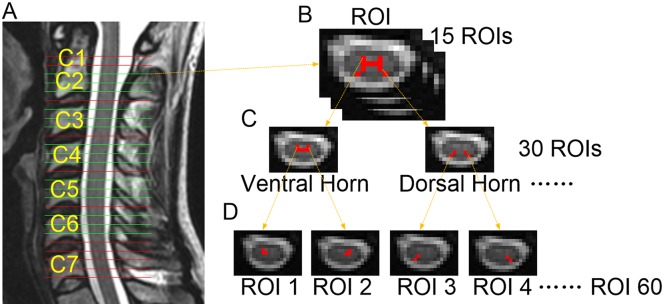
Gray matter regions of interest (ROIs) definition in the cervical spinal cord. The rsfMRI scanning field of view (FOV) and slice location that cover vertebrae C1 to C7 (a); the gray matter was drawn on post-processed echo planar imaging (EPI) images (b); the gray matter ROI was then delineated and split into ROI1 (ventral horn) and ROI2 (dorsal horn) (c); left and right parts of each horn were also furtherly separated; finally 60 ROIs were extracted from each subject (d).

Region of interests (ROIs) were manually drawn on 15 out of 26 slices from the C2 to C6 segments to delineate the grey matter (3 slices for each segment) based on comparatively higher image contrast and anatomy of spinal cord gray matter. Slices crossing every inter-vertebral disc within the C1 and C7 segments were discarded due to FOV mismatch or severe artifact. The gray matter ROI was split into ROI1 (ventral horn) and ROI2 (dorsal horn) according to their function because dorsal horn and ventral horn are involved in sensory and motor neural information respectively. Then ventral and dorsal horn ROIs were furtherly separated into left and right parts. In total, 60 ROIs were drawn in 5 segments (3 slices per segment and 4 ROIs per slice, Fig 1).

### Amplitude of low frequency fluctuation (ALFF) analysis

ALFF was calculated through REST toolbox under MATLAB. The REST toolbox will calculate the square root of the power spectrum of BOLD signals from each voxel and compute the sum of frequencies in the low frequency band (0.01–0.08Hz) [22]. For each ROI, the ALFF was determined through calculating the mean value of ALFF values of all voxels in this ROI.

### Statistical Analysis

Two sample t-test was used to investigate the differences of ALFF among healthy subjects and CSM patients. In order to evaluate the relationship between ALFF and clinical symptoms, CSM patients were divided into two groups based on their JOA scores, severe group (JOA<13, 10 patients, age = 67±7 years, age ranged from 59 to 83 years) and mild group (JOA≥13, 8 patients, age = 49±14 years, age ranged from 26 to 67 years). Two sample t-test was performed to compare differences of APCR and ALFF between mild CSM patients and severe CSM patients. The ALFF comparison was conducted on the cervical cord and segments from C2 to C6 separately. Mean value of all ROIs in 3 slices of one segment (3 slices * 4 ROIs = 12 ROIs) was used for statistical analysis. ALFF values of all ROIs (5 segments * 3 slices * 4 ROIs = 60 ROIs) were averaged to be one ALFF for whole cord.

## Results

### Healthy subjects vs CSM patients

**Fig 2:**
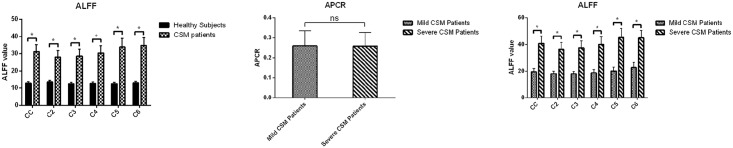
ALFF comparison between healthy subjects and CSM patients. In the whole cervical cord (CC) and each segment from C2 to C6, amplitude of low frequency fluctuation (ALFF) had the trend of healthy subjects < CSM patients. From two sample t-test results, all of the differences were significant (a). After separating CSM patients into mild (JOA≥13, 8 patients, age = 49±11 years, age ranged from 26 to 67 years) and severe (JOA<13, 10 patients, age = 67±7 years, age ranged from 59 to 83 years) groups, Anterior-Posterior Compression Ratio (APCR) of each patients in mild and severe groups was compared. The difference of APCR between mild and severe CSM patients was not significant (b). ALFF had the trend of mild CSM patients < severe CSM patients. From two sample t-test results, all of the differences were significant (c) (*: P<0.05, significant, ns: P>0.05, not significant).

ALFF values of healthy subjects and CSM patients on the cervical cord and segments from C2 to C6 are separately listed in Table 1. The ALFF comparison result is shown in Fig 2a. In the whole cervical spinal cord and each segment, the ALFF values of healthy subjects were less than those of CSM patients. Two sample t-test results indicated that all the differences of ALFF between healthy subjects and CSM patients were significant.

**Table 1:** ALFF values of healthy subjects and CSM patients (mean ± SEM).

ALFF values	Healthy subjects (N = 25)	CSM patients (N = 18)	F	t	P
CC	12.90 ± 0.83	31.23 ± 4.12	28.612	-4.355	<0.001
C2	13.58 ± 0.92	28.07 ± 3.64	27.715	-3.858	0.001
C3	12.60 ± 0.83	28.71 ± 3.82	32.995	-4.117	0.001
C4	12.70 ± 0.85	30.42 ± 4.20	30.238	-4.135	0.001
C5	12.55 ± 0.85	34.02 ± 4.93	23.241	-4.290	<0.001
C6	13.11 ± 0.95	34.91 ± 4.39	30.527	-4.849	<0.001
ALFF values	Mild CSM patients (N = 8)	Severe CSM patients (N = 10)	F	t	P
CC	19.38 ± 2.55	40.70 ± 5.59	3.983	-3.188	0.006
C2	17.87 ± 1.95	36.24 ± 5.10	4.686	-3.362	0.006
C3	17.83 ± 1.75	37.41 ± 5.37	8.639	-3.465	0.005
C4	18.12 ± 2.65	39.87 ± 5.77	4.243	-3.074	0.007
C5	20.04 ± 2.91	45.20 ± 6.79	4.772	-3.407	0.005
C6	22.55 ± 3.81	44.80 ± 5.67	2.141	-3.081	0.007

Note: CC—whole cervical cord; C2/C3/C4/C5/C6—C2/C3/C4/C5/C6 segment of cervical cord

### Mild CSM patients vs severe CSM patients

After grouping patients according to the JOA score, APCR was compared between the two groups of CSM patients and the results are listed in Fig 2d. There was no significant difference in APCR between mild CSM (0.259±0.075) and severe CSM groups (0.258±0.067) (F = 0.034, t = -0.020, P = 0.985). Mild and severe CSM groups had no significant difference in APCR.

ALFF values of mild and severe CSM patients on the cervical cord and segments from C2 to C6 separately are listed in Table 1. The ALFF comparison result is shown in Fig 2c. In the whole cervical spinal cord and each segment, the ALFF values of mild CSM patients were significantly less than those of severe CSM patients.

## Discussion

RsfMRI showed that ALFF was increased in CSM patients when compression appeared on the cervical cord. Furthermore, severity of clinical symptom in CSM was associated with ALFF changes. Patients with higher JOA score presented lower ALFF. Vice versa, patients with lower JOA score presented higher ALFF. This finding could help understand the mechanism of CSM neural development.

On the one hand, the severity of compression is usually measured in T2-weighted MRI images with anterior-posterior transverse diameter [23]. APCR is a parameter delineating the deformity and flattening of the cervical cord and it is calculated through dividing the sagittal diameter of the transverse diameter of the axial section of T2W images [24–28]. On the other hand, the severity of clinical symptom is commonly evaluated by JOA in clinical setting [29], in comparison with other evaluation tools like Nurick scale, Harsh scale, Cooper scale, Prolo scale and European Myelopathy score [30–34]. CSM patients in this study were recruited with similar degrees and patterns of compression to the cervical cord but with different JOA scores, i.e., 14.5±1.6 in 8 patients and 10.7±2.1 in 10 patients. The different clinical presentations of JOA in this group of patients can exclude the effect of compression ratio on the severity of clinical symptom. Spinal cord rsfMRI was applied to investigate the potential explanation of intrinsic functional organization between different clinical symptom presentations.

In rsfMRI study, ALFF is used as a metric to measure the level of regional functional neural activity [20]. In this study, CSM patients have higher ALFF values than healthy subjects. Higher ALFF values indicate the high level of regional neural activity in the spinal cord. During resting state, brain regions with ALFF values higher than the global mean level exhibit significantly a higher metabolic rate of oxygen and glucose than other regions [22], which means that those regions have higher level of neural activity. When there is an injury to the spinal cord, rsfMRI study has confirmed that the processing of nociceptive and tactile inputs engages a much more complex spinal cord circuitry than previously recognized [16]. As a result, it will add extra burdens to neurons in the spinal cord and make neurons consume more energy to maintain the function of the neural network. Taken together, for CSM patients, the level of neural functional activity rises as reflected by the increased ALFF values.

CSM patients with stenosis on the spinal cord canal may have various neurological function deficits [3]. However, results of this study demonstrated that ALFF values can be well associate with the neurological function deficits. Therefore, it can be assumed that ALFF is a potential new valuable factor to reflect various neurological function deficits under similar stenosis conditions.

Based on our findings, the phenomenon that the spinal cord increased its neural activity level in response to compression of CSM coincides with the existed concept of neural plasticity that the nervous system can change its function in response to injury [35]. Previous anatomical studies have reported that neural plasticity occurs in the nervous system after spinal cord injury [36, 37]. Besides, intrinsic brain functional plasticity was also reported in CSM patients that the functional connectivity strength increased after CSM happened [38]. To our best knowledge, our study is the first demonstration of intrinsic functional plasticity on the spinal cord in CSM patients, and provides a possible explanation for neural development mechanism of CSM.

In addition, F-test between healthy subjects and CSM patients showed large F values which surpassed the cutoff F value (cutoff F value 2.20, degree of freedom [df] of larger variation group was 12, df of smaller variation group was 23) (Table 1). The large F values indicated that CSM patients had significantly larger ALFF variations than healthy subjects. CSM patients are usually with more complex neural activity conditions than healthy controls, corresponding to their various symptoms and conditions [39–41]. Since ALFF is the most reliable and reproducible rsfMRI parameter in reflecting regional functional neural activity, there should be more ALFF variations in CSM patients than in healthy subjects.

In clinical practice, therapy management for CSM usually contains two strategies: conservative and surgical treatment [42, 43]. The ability of neural plasticity should be considered to determine whether a patient with CSM to receive surgical treatment without further delay of natural progress of neurological symptom development. This ALFF technique can show the functional plasticity of CSM patients, and suggests the significant time point to perform surgical treatment with risk of missing the most appropriate intervention time. It may provide a new indication for surgeons to select the surgical time for curing spinal cord function of CSM.

The fractional anisotropy (FA) value of diffusion tensor imaging (DTI) is another MRI technique that has already been applied in the clinical investigation of CSM patients. DTI focuses on the ultrastructural information of white matter of central nervous system (CNS) [44]. ALFF is a technique that focuses on the functional activity of gray matter of CNS [18]. Therefore, the combination of DTI and ALFF may provide comprehensive understanding of the mechanism behind CSM [45]. The scanning for rsfMRI and DTI have no conflict with each other, which makes both scanning possible to be co-utilized in detection. More importantly, the co-utilized DTI and ALFF may provide a comprehensive evaluation in both structure and function of the spinal cord, providing precise diagnosis of CSM for treatment decision making.

It is a technical challenge to reduce physiological noise in spinal cord rsfMRI, including periodic motion and hemodynamic washout [46–48]. During cardiac and respiratory cycles, periodic pulsatory motion of both CSF and spinal cord itself produces degradation on spinal cord signal [49]. Motion of the spinal cord can be observed during scanning, with a maximum displacement of approximately 0.5-1mm [46]. In the present study, the subjects were carefully recruited to have a good compliance to this study. They were asked to lie on the bed peacefully, with comfortable bed and sponge filling to avoid their motion. In the processing of rsfMRI, the maximum tolerance of displacement is below half of the voxel size in each direction, which is the same as commonly used tolerance in rsfMRI brain studies. The periodic signal from CSF was decreased through a band pass filter of 0.01–0.08Hz because the frequency of periodic changes of CSF flow with the cardiac cycle is about 1Hz [46]. To reduce the impact from hemodynamic washout caused by large vessels [50], the flow contribution to the BOLD signal was minimized by the fast imaging speed so that one slice was acquired less than 80ms in an interleaved way. Also, the application of nuisance regression helped to regress out physiology signals from time series of rsfMRI images.

**Fig 3:**
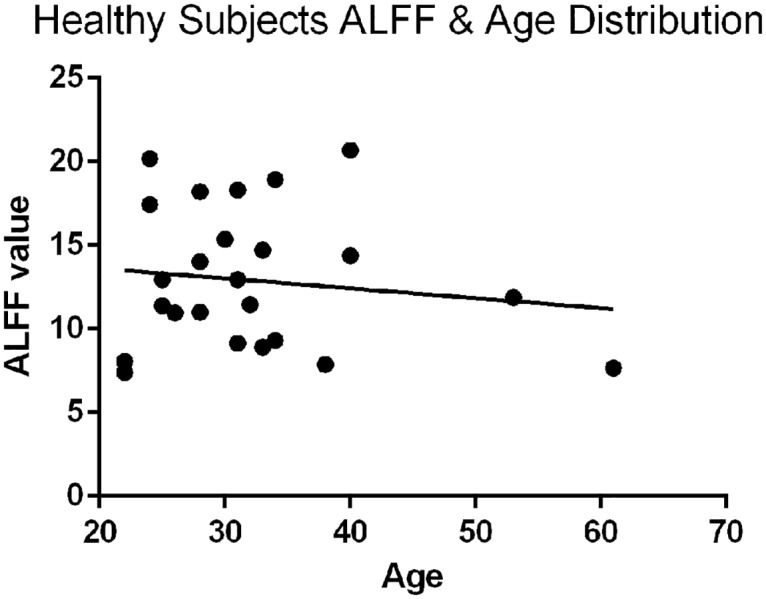
Linear regression between age and ALFF values in healthy subjects. The linear regression result showed that ALFF was not affected by age.

There are some limitations in the current study. It should be noted that it is difficult to recruit an age-matching group because most of elderly healthy subjects would have more or less degeneration of the cervical spine. So the conclusion of this study may not represent the full range of population. However, linear regression between age and ALFF values of healthy subjects showed that ALFF was not affected by age (Fig 3). Besides, the cross-sectional diameter of the spinal cord is very small (10-15mm), which means that the MRI images of the spinal cord have a limited number of voxels [49]. MRI of the spinal cord also involves considerable magnetic field inhomogeneity because of the large susceptibility differences among tissues. In this study, to observe the functional organization of the spinal cord integrally, we manually draw ROIs on the gray matter of the cervical spinal cord from C2 to C6 segments. This manually ROI definition method does have advantages and disadvantages. Although drawing ROIs manually is time-consuming, labor-intensive and less objective when compared to obtaining ROIs from normalized images, it can guarantee the accuracy of ROI location on the gray matter of the spinal cord, with high inter-observer and intra-observer reliability.

## Conclusions

This study revealed the increase of ALFF in the gray matter of the cervical spinal cord after CSM. The severity of clinical symptom in CSM is likely associated with the degree of ALFF increase. The novel spinal cord rsfMRI investigation facilitates the understanding of the mechanism of neurological development of CSM. It may promote future researches to improve diagnosis, prognosis and management of CSM.
